# Dental versus Medical Education

**Published:** 1906-06

**Authors:** Tennyson Ricard

**Affiliations:** Chicago, U. S. A.


### DENTAL VERSUS MEDICAL EDUCATION.

By Tennyson Ricard, B. S., D. D. S., Chicago, U. S. A.

Much has been written and said about the great advancements made in dental science within the last decade, and yet while there is much to be proud of in the scientific and professional standing of dentistry to-day, there is still so much need for improvement and broadening out in this great field of needed activity, that we may truthfully say "the profession is still in its mere infancy."

No intelligent person, competent to judge, will refute the assertion that "the practice of dentistry is just as much the practice of a branch or specialty of general medicine as is the practice of the eye, ear, throat, etc." Therefore, if the specialist who limits his practice to diseases of the eye, ear, skin, etc., must be a qualified general physician first, why should not the dentist, who is also a specialist devoting his attentions to special and very important organs of the same human body, be equally trained and also a general physician before taking up his special study and practice?

When dentistry was merely a sort of skilled-mechanical trade, and its votaries were not expected to administer and understand the use of stimulants, tonics, escharotics, anesthetics, etc., or perform various oral operations requiring the internal use of powerful or dangerous medicinal agents, it was unnecessary for them to possess a definite knowledge of the action of drugs.

With the gradual enlightenment of the general public in the possibilities accomplished by dental science, the field of practice will rapidly widen, and the D. D. S. will be expected to nearer equal the M. D. in general medical and physiological education.

The dentist who knows best his anatomy, pathology, therapeutics, medicine, etc., understands the exact application of this knowledge. This same dental doctor is competent to advise the soon-to-be mother in the proper care of her teeth; prescribe the best diet and other medication that will result in the production of perfect teeth for her future babe; give systemic treatment to improve the structure of chalky, soft teeth; check the spread of dental erosions; permanently cure abcesses and pyorrheal conditions, facial neuralgia, etc.

This modern dentist is patronized by a class of patients who prefer to pay for *advice, consultation,* and *preventive*

*treatment* than to contend with the numerous ailments resulting from a neglected condition of the *grinding-stones* of the *human mill.*

I wish to ask a question that has occurred to my mind as the result of lectures before classes at the medical colleges as well as through the developments of my own private practice: "If dentists understand and devote their attentions to the mouth, which is the gateway to the digestive tract, why should they not thoroughly understand and treat all forms of diseases of this same entire tract?

Few medical doctors will refute the assertion that about 90% of all alimentary disorders, such as indigestion, dyspepsia, constipation, mal-nutrition, gastric and intestinal disorders, have their origin in improper feeding and faulty mastication. The dentist should be the doctor to attend such ailments, and thus further broaden his field of practice and usefulness.

That this branch of therapeutics should be added to the dentist's work, is evidenced by the fact that very few physicians, in battling with alimentary troubles, ever think of the origin of most alimentary disorders—the teeth. They seem to know nothing of the importance of the dental organs; they have not had the lectures and instructions that all physicians should have on dental diseases, oral hygiene, and dental substitutions. They need to be told a thing or two about dentistry and these things should be told them by a competent dentist who knows.

In deciding upon entering an institution to prepare for the study of dentistry, students should go where broad and modern dental knowledge can be secured. This knowledge can best be obtained wherever the training for the first two years is the same for both dental and medical students—a condition which is now being established in one or two Chicago institutions.

The great advantages to be derived from such a combined system of college work can be appreciated by practicing dentists who had no such opportunities themselves while at college, where the courses of instruction in chemistry, medicine, anesthetics, diagnosis, etc., were more conspicuous in the college catalogs than in real class work.

It is evident that there is need of decided expansion of the present confines of dental practice if we all must find success in our profession, and the yearly floods of graduates do not abate. By adding to dental science what properly belongs to its scope and practicing all branches of healing that should be attended by the dental doctor, we will have a vocation as highly dignified, as lucrative and more pleasant to follow than general medicine.

With reference to the subject of drugs or medicine, how many dentists in ten can write a simple prescription for neuralgia? We know not one in every ten is able to do this, or even able to make the simplest solution of cocaine for a local anesthetic. Should collapse happen his patient in the chair, that they know so little of medicine and its action, and their title of doctor is a sort of "make-believe" handle to their names.

That the dentist should have the knowledge of the average physician in reference to medicine, pathology, etc., is admitted, and therefore the place to get this is alongside of the medical student in the same class rooms. All the scientific branches in dental and medical colleges could and should be taken together by the students of both professions.

